# Two-Generation Genetic Evaluation of Female Reproductive Performance in Pacific White Shrimp (*Penaeus vannamei*) Under SPF Conditions

**DOI:** 10.3390/ani16020235

**Published:** 2026-01-13

**Authors:** Jiaqi Yu, Jie Kong, Sheng Luan, Jiawang Cao, Mianyu Liu, Kun Luo, Jian Tan, Ping Dai, Zhaoxin Wang, Juan Sui, Xianhong Meng

**Affiliations:** 1 College of Fisheries and Life Science, Shanghai Ocean University, Shanghai 201306, China; yujiaqi115622@163.com; 2 State Key Laboratory of Mariculture Biobreeding and Sustainable Goods, Yellow Sea Fisheries Research Institute, Chinese Academy of Fishery Science, Qingdao 266071, China; kongjie@ysfri.ac.cn (J.K.); luansheng@ysfri.ac.cn (S.L.); caojw@ysfri.ac.cn (J.C.); 2022213007@stu.njau.edu.cn (M.L.); luokun@ysfri.ac.cn (K.L.); tanjian@163.com (J.T.); daiping54@163.com (P.D.); 3 Laboratory for Marine Fisheries Science and Food Production Processes, Qingdao Marine Science and Technology Center, Qingdao 266237, China; 4 BLUP Aquabreed Co., Ltd., Weifang 261311, China; wangzhaoxin2021@126.com

**Keywords:** *Penaeus vannamei*, reproductive traits, heritability, genetic correlation, genetic gain, SPF

## Abstract

Reproductive inefficiency remains a major constraint in *Penaeus vannamei* hatcheries due to high rates of non-spawning females. This study presents the first two-generational quantitative genetic analysis of female reproductive performance under standardized SPF (Specific Pathogen-Free) conditions. A total of 986 females across two generations (2021–2022) from 198 full-sib and 68 half-sib families were evaluated. Traits analyzed included spawning frequency (SF), mean spawning interval (MSI), number of eggs laid for the first time (NE1), average spawning (AS), total spawning (TS), and spawning success (SS). Heritability estimates for SF, SS, and TS were moderate (0.30 ± 0.06, 0.23 ± 0.06 and 0.28 ± 0.07, respectively), while MSI, NE1, and AS showed low heritability (0.10–0.16). When analyzed separately by year, heritability estimates declined substantially for most traits in the second generation. Strong positive genetic correlations were observed between SF, MSI, NE1, AS, and TS, with pairwise estimates ranging from 0.82 to 0.99, indicating that these traits are under shared genetic control. Despite not being direct selection objects, all reproductive traits exhibited relative genetic progress (246–488% per generation), which is attributable to the high selection intensity applied to the parental generation. Our findings provide a robust foundation for integrating reproductive performance into breeding programs for *P. vannamei*, particularly under biosecurity and commercial feed-dominated conditions.

## Simple Summary

This study estimated genetic parameters for female reproductive traits in *Penaeus vannamei* under SPF (Specific Pathogen-Free) conditions across two consecutive generations, using a pedigree-based best linear unbiased prediction (pBLUP). The analyzed traits included spawning frequency (SF), mean spawning interval (MSI), number of eggs laid for the first time (NE1), average spawning (AS), total spawning (TS), and spawning success (SS). Our findings confirm that female reproductive traits in *P. vannamei* are heritable and can be effectively improved through selective breeding. Although heritability declined for some traits in the second year, core reproductive traits consistently exhibited low to moderate heritability and shared common genetic control. These results provide new insights into the feasibility of selecting for enhanced reproductive performance without compromising other economically important traits.

## 1. Introduction

The Pacific white shrimp, *Penaeus vannamei*, is the most widely cultured penaeid species globally in terms of production volume [1]. Since its introduction to China in the late 1980s, *P. vannamei* has become the dominant species in domestic aquaculture [2,3]. China now contributes roughly one-third of global farmed shrimp output and annually consumes about 1 million broodstock pairs to produce approximately 1.5 trillion post-larvae [4]. However, reproductive inefficiency remains a bottleneck in *P. vannamei* hatcheris. Field observations and multiple studies have consistently reported that a significant proportion of mature females either fail to spawn or spawn only once, with non-spawning rates ranging from 14% to over 50% [5,6,7,8]. This low and uneven reproductive output not only limits nauplii yield, but also increases broodstock maintenance costs.

Reproductive traits such as spawning frequency (SF) and total spawning (TS) are economically critical [9]. Yet these traits are often overlooked in breeding programs that prioritize growth or disease resistance [10,11,12]. Due to the species’ high fecundity, this neglect can lead to a few high-performing broodstock dominating offspring production, thereby reducing effective population size and eroding genetic diversity. Consequently, understanding the genetic architecture of reproductive traits—including their heritability, genetic correlations, and genetic progress—is essential for designing balanced breeding strategies that sustain both productivity and reproductive performance.

Previous studies have estimated heritability for reproductive traits in *P. vannamei* with varying results. For instance, Arcos [13] reported heritability estimates of 0.09–0.15 for SF, while Ren [14] estimated moderate heritability (0.15 ± 0.06) and high genetic correlations between body weight and egg number (0.93 ± 0.10). Caballero-Zamora [15] found low heritability for number of nauplii (0.03 ± 0.04) but a positive genetic correlation (0.49 ± 0.15) between female body weight and egg number. Related investigations in other aquaculture species have reported comparable quantitative-genetic parameters. Gonçalves [16] estimated the heritability of SF in *Oreochromis niloticus* to be 0.15 ± 0.09. Wu [17] documented a heritability of 0.32 ± 0.08 for TS in *Crassostrea gigas* and further showed that the genetic and phenotypic correlations between female reproductive traits and growth traits were low, ranging from −0.44 to 0.33. Wang [18] found that TS in the *Exopalaemon carinicauda* had a heritability of 0.45–0.52, whereas the heritability of age at first spawning ranged from 0.22 to 0.77. Macbeth [19] reported that the heritability estimates for days to spawn, egg number, and nauplii number in black tiger shrimp were 0.47 ± 0.15, 0.41 ± 0.18, and 0.27 ± 0.16, respectively. These studies provide a basis for the genetic improvement of reproductive traits but were mostly limited to single-generation datasets or lacked control for environmental confounders such as mating system or feeding regime.

To date, no study has systematically evaluated the genetic parameters of reproductive traits across multiple generations under standardized SPF (Specific Pathogen-Free) conditions. While SPF environments—now standard in many breeding programmes—effectively minimise disease risk, they do not directly address reproductive failure [20,21]. Currently, broodstock maturation in shrimp still largely relies on fresh feeds such as squid and polychaetes, which can introduce pathogens into production systems. To mitigate this issue, we developed a pelleted feed based on optimized nutritional formulations that support gonad development in broodstock [22], supplemented with a controlled amount of fresh-chilled squid. All feeds were subjected to rigorous nutritional and pathogen testing prior to use, ensuring they meet the requirements for gonad development while avoiding the risk of pathogen contamination associated with fresh feeds. After confirming the efficacy of the formulated feed in promoting broodstock gonad maturation, we analyzed two consecutive generations (2021–2022) from the BLUP Aquabreed Co., Ltd. (Weifang, China) breeding nucleus, whose breeding objectives were WSSV (white spot syndrome virus) resistance and growth traits.

This study aims to fill that gap by estimating heritability, genetic correlations, and genetic progress for key reproductive traits in *P. vannamei* across two consecutive generations (2021–2022) under a controlled breeding environment with natural mating and standardized nutrition. Our findings will provide novel insights into the feasibility of selecting for improved reproductive output without compromising other economically important traits.

## 2. Materials and Methods

### 2.1. Population Structure

**Figure 1:**
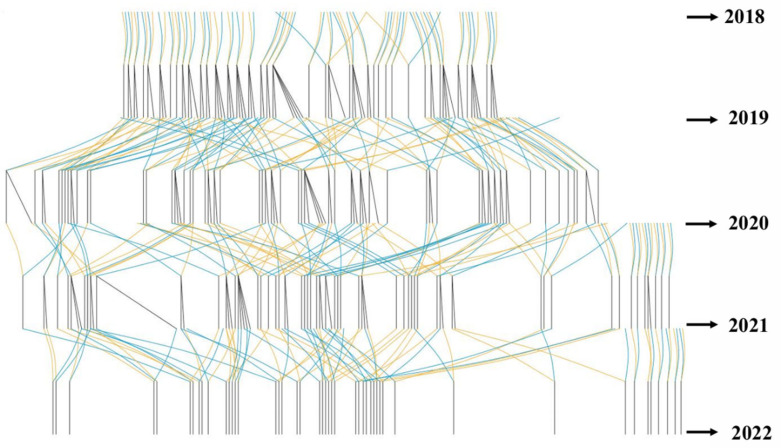
Pedigree Structure Chart of the Maternal in the Breeding Nucleus. Yellow lines represent female parents (dams), blue lines represent male parents (sires), and black lines represent the newly formed families in each generation.

The counts of females, sires, dams, and full- and half-sib families for 2021–2022 are listed in Table 1, with the pedigree chart displayed in Figure 1.

The breeding program was initiated in 2018 at BLUP Aquabreed Co., Ltd. (Weifang, Shandong, China). The base population comprised two introduced lines: Florida (2018) and Ecuador (2020). The Ecuadorian line is widely recognized in commercial production for its strong disease resistance and good environmental adaptability, whereas the Florida line is generally characterised by favourable growth performance. These two lines were crossed to generate progeny expected to combine robustness and growth, which served as the base population for the present study. Pedigrees of founders were unknown. A multi-trait selection scheme was applied targeting WSSV resistance and growth performance. The inbreeding rate was controlled below 0.8% per generation. Families were established using a single-parent nested mating design, where one or two females from different families were paired with fifteen males from a third family [10,23].

### 2.2. Broodstock Management and Reproductive Data Collection

All procedures were conducted in strict accordance with the SPF conditions established at BLUP Aquabreed Co., Ltd. (Weifang, China). At 10 months of age, candidate females were transferred to indoor maturation tanks (16 m^2^, cement-concrete, 6–7 shrimps m^−2^). The rearing environment was maintained at 28 ± 1 °C, 31 ± 1 ppt salinity, pH 8.0–8.2, total ammonia-N < 0.5 mg L^−1^, and nitrite-N < 0.05 mg L^−1^. A 75% daily water exchange was maintained. Fifteen healthy male shrimp were randomly selected from each family and temporarily reared in 3 m^2^ concrete tanks. Except for temperature (27 ± 1 °C), all other rearing conditions were consistent with those of the female shrimp.

Females received a mixed diet for 20 days. This consisted of fresh-chilled squid (10% BW day^−1^, offered in two equal meals) and a formulated maturation pellet (56% crude protein, 11% crude lipid; 4% BW day^−1^, offered in four equal meals). All feeds were rigorously tested prior to use. Nutritional standards were verified, feeds were confirmed free of specific pathogens before being approved for application. Pathogen screening included WSSV, Taura Syndrome Virus (TSV), *Vibrio parahaemolyticus (Vp_AHPND_)*, Yellow Head Virus (YHV), and Infectious Hypodermal and Hematopoietic Necrosis Virus (IHHNV). Thereafter, unilateral eyestalk ablation was performed under ice anesthesia to induce ovarian development. After a subsequent 20-day recovery period, ovarian staging was carried out daily at 8:00 using a non-invasive visual index based on size, color and opaqueness [24,25]. Ovarian development was classified into four stages according to the criteria of Tinikul [26]. Females reaching ovarian stage IV—a recognized indicator of full maturity—were selected and transferred to 3 m^2^ male tanks according to a mating scheme. Mating was conducted through natural fertilization. Each evening at 20:00, females were checked for the presence of spermatophore on the thelycum. Successfully mated females were gently netted and transferred to a 180 L spawning tank individually. The next morning, spawned females were returned to their original tanks.

Spawning activity was monitored continuously for 48 days in both 2021 and 2022 cycles. Each spawning event was time-stamped and eggs were collected in 170 L hatching buckets. Total egg number was estimated using a sampling and counting method. Specifically, the water in the spawning bucket was stirred to distribute the eggs evenly. Next, 3 mL samples of egg-bearing water were collected from the mid-depth of the bucket using a pipette, and the average number of eggs in these samples was calculated. The total spawning output was then estimated based on the principle of proportional scaling. The following reproductive traits were recorded for every female:

- Spawning frequency (SF): total number of spawning events by females during a 48-day spawning period.
- Mean spawning interval (MSI): average interval (days) between consecutive spawns.
- Number of eggs laid for the first time (NE1).
- Total spawning (TS): cumulative egg number over the entire 48-day observation period.
- Average spawning (AS): total spawning output/spawning frequency.
- Spawning success (SS): whether the female shrimp spawned.

Water-quality parameters were monitored twice daily. The entire breeding process, including mating, involved daily sampling of broodstock, aquaculture water, and larvae. These sampling and monitoring protocols were conducted to ensure the absence of target pathogens (TSV, WSSV, *Vp_AHPND_*, YHV, and IHHNV).

### 2.3. Statistical Analysis

#### 2.3.1. Data Preparation

All raw data were first imported into Microsoft Excel 2017 for initial cleaning and descriptive statistics (means, minima, maxima, standard deviations, coefficients of variation). Records with obvious data-entry errors were removed.

#### 2.3.2. Genetic Parameters

Single-trait animal models were fitted with ASReml 4.0 [27] to estimate variance components and heritabilities for SF, MSI, NE1, AS and TS. Due to the limited sample size, only the year was included as a fixed effect in the model, with no environmental covariates added. The model was:

$$y_{ij}=\mu +{Year}_{j}+a_{i}+e_{ij}$$

where $y_{ij}\;$ is the target traits of the ith individual; μ  is the overall mean; ${Year}_{j}$ is the fixed effect of the  jth year; $a_{i}$ is the additive genetic effect of the ith shrimp; and $\;e_{ij}$ is the random residual error of the ith individual.

Convergence failure occurred when a common full-sib (family) effect was added, owing to the limited number of females per family and the single-sire nested mating design. Consequently, the common-environment term was omitted. Narrow-sense heritability was derived as $h^{2}=\sigma_{a}^{2}/(\sigma_{a}^{2}+\sigma_{e}^{2})$.

A sire-dam and probit model was used to estimate the heritability of SS. The model was constructed as described in ASReml 4.0 [27].

$$\lambda_{i}=\mu +{Sire}_{j}+{Dam}_{j}+e_{ij}$$

$$y_{i}=\left\{\begin{array}{c} 0,\;if{\;\lambda }_{i}\leq 0\\ 1,\;if{\;\lambda }_{i}>0 \end{array}\right.$$

where $y_{i}$ is the spawn status (1 = spawning success, 0 = non-spawning success) of the ith shrimp; ${\;\lambda }_{i}\;$ is the underlying liability of $y_{i}$ which is assumed to be a cumulative standard normal distribution; μ is the overall mean; ${\;Sire}_{j\;}$ and ${Dam}_{j}$ are the additive genetic effects of the jth sire and dam, which is represented as  Sire or $Dam\sim \left(0,{A\sigma }_{sd}^{2}\right)$, where $\;\sigma_{sd}^{2}=\sigma_{s}^{2}=\sigma_{d}^{2}$ and A is the additive genetic relationship matrix among all shrimps; and $e_{j}\;$ is the random residual error of the ith individual, with $e\sim \left(0,\;{I\sigma }_{e}^{2}\right)$. The residual variance of λ was assumed to be 1. The common environment effect was excluded from this model because it could not be partitioned effectively, likely due to the limited genetic connections between families. The phenotypic variance was the sum of ${2\sigma }_{sd}^{2}$ and $\sigma_{e}^{2}\;(\sigma_{p}^{2}={2\sigma }_{sd}^{2}+\sigma_{e}^{2})$. Heritability ($h_{x}^{2}$) was computed as the ratio between ${4\sigma }_{sd}^{2}$ and $\sigma_{p}^{2}(h_{x}^{2}={4\sigma }_{sd}^{2}/\sigma_{p}^{2})$.

#### 2.3.3. Genetic Correlation

Pairwise bivariate animal models (same fixed and random structure as above) were used to estimate genetic (*r_g_*) correlations among SF, MSI, NE1, AS and TS.

$$r_{gij}=cov\left(\sigma_{i},\sigma_{j}\right)/\sigma_{ai}\sigma_{aj},$$

where $r_{gij}$ is the genetic correlations between trait i and trait j; $cov(\sigma_{i},\sigma_{j})$ is the covariance between trait i and trait j; and $\sigma_{ai}$ and $\sigma_{aj}\;$ are the additive genetic standard deviations for trait i and trait j, respectively.

#### 2.3.4. Significance Analysis of Differences

Approximate standard errors for *h*^2^ and *r_g_* were obtained from the inverse of the average information matrix. *Z*-tests [28] evaluated whether *h*^2^ differed from zero and whether *r_g_* differed from unity:

$$Z=(X_{i}-X_{j})/\sqrt{\sigma_{i}^{2}+\sigma_{j}^{2}},$$

where $X_{i}\;$ and $X_{j}\;$ are the estimates of heritability or genetic correlation; while $\sigma_{i}^{2}$ and $\sigma_{j}^{2}\;$ represent the standard errors of the corresponding estimates of heritability and correlation coefficients. When testing whether the heritability significantly differs from 0, $X_{j}\;$ and $\sigma_{j}$ are both defined as 0; when testing whether the correlation coefficient significantly differs from 1, $X_{j}\;$ and $\sigma_{j}\;$ are defined as 1 and 0, respectively.

#### 2.3.5. Genetic Gain

Best linear unbiased predictions (BLUP) of breeding values (EBVs) were obtained for all animals using the single-trait model. Genetic gain per generation was calculated as:

$$\Delta G=(\mathrm{mean}\;{\mathrm{EBV}}_{\mathrm{2022}}-\mathrm{mean}\;{\mathrm{EBV}}_{\mathrm{2021}})/\mathrm{mean}\;{\mathrm{EBV}}_{\mathrm{2021}}\times \mathrm{100}\%$$

## 3. Results

### 3.1. Descriptive Statistics

**Figure 2:**
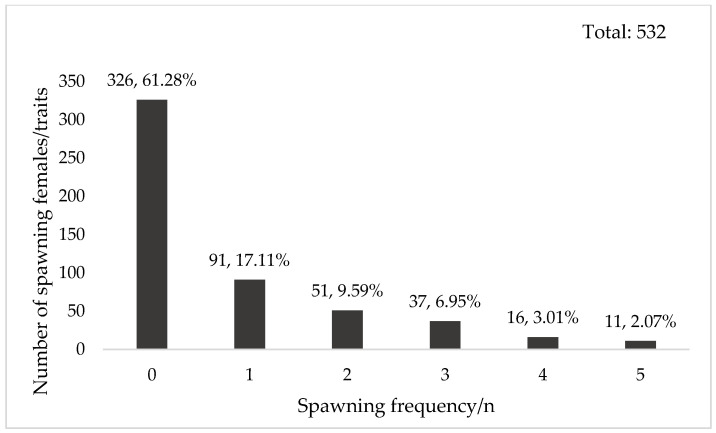
Spawning frequency of 2021 generation. Note: The numbers above the bars indicate the number of individuals for each spawning frequency and the corresponding percentage of the total.

**Figure 3:**
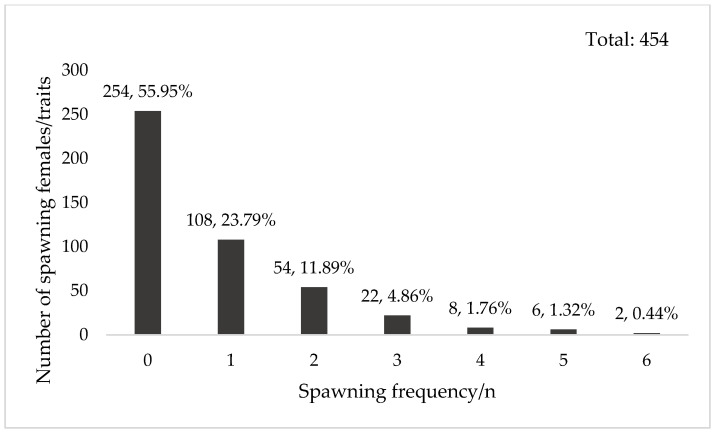
Spawning frequency of 2022 generation. Note: The numbers above the bars indicate the number of individuals for each spawning frequency and the corresponding percentage of the total.

Across two consecutive generations (2021–2022), 986 females from 198 full-sib families were monitored for 48 days. In 2021, SF ranged from 0 to 5; in 2022, it ranged from 0 to 6. The proportion of females that failed to spawn was 55.95% in 2021 and 61.28% in 2022. Among spawning individuals, 1–3 spawns were most common (33.65–40.54%), while fewer than 6% exhibited more than 3 spawns (Figure 2 and Figure 3).

Mean values and coefficients of variation (CV) for reproductive traits are summarized in Table 2. MSI exhibited the highest variability (CV = 230.53% in 2021; 254.7% in 2022), followed by TS (CV = 168.52% in 2021; 138.32% in 2022).

### 3.2. Variance Components and Heritability

The variance components for all reproductive traits are presented in Table 3.

In 2021, all traits exhibited medium to high heritability estimates. Specifically, SF, NE1, AS, and SS exceeded 0.30, with SS reaching as high as 0.79. MSI and TS demonstrated moderate heritability, ranging from 0.22 to 0.28. However, heritability estimates declined substantially in 2022. SF fell to 0.19 ± 0.08, TS to 0.18 ± 0.07, and SS to 0.12 ± 0.08, while MSI, NE1, and AS dropped to 0.06–0.10.

Across both generations (2021–2022), heritability estimates ranged from low to moderate: SF 0.30 ± 0.06, MSI 0.10 ± 0.04, NE1 0.12 ± 0.05, AS 0.16 ± 0.06, TS 0.28 ± 0.07, and SS 0.23 ± 0.06. All estimates were significantly greater than zero (*p* < 0.05). The decline in heritability for most traits in 2022 reflects decreased additive variance caused by intense directional selection.

### 3.3. Genetic Correlations

Genetic and phenotypic correlations among SF, MSI, NE1, AS, and TS were estimated across both generations (Table 4). Genetic correlations ranged from 0.82 ± 0.10 to 0.99 ± 0.00. The strongest associations were observed between NE1 and AS (*r_g_* = 0.99 ± 0.00), followed by NE1 and TS (*r_g_* = 0.99 ± 0.05), AS and TS (*r_g_* = 0.98 ± 0.04), SF and NE1 (*r_g_* = 0.83 ± 0.11), SF and AS (*r_g_* = 0.82 ± 0.10), and SF and TS (*r_g_* = 0.93 ± 0.02). MSI exhibited near-perfect positive genetic correlations with NE1 (*r_g_* = 0.99 ± 0.18), AS (*r_g_* = 0.94 ± 0.15) and TS (*r_g_* = 0.99 ± 0.08). Phenotypic correlations were consistently positive and moderate to high (*r_p_* = 0.64–0.91). The highest phenotypic correlation was between SF and TS (*r_p_* = 0.91 ± 0.00), followed by NE1 and AS (*r_p_* = 0.96 ± 0.00), NE1 and TS (*r_p_* = 0.77 ± 0.01), and AS and TS (*r_p_* = 0.80 ± 0.01). All genetic correlations were significantly different from zero (*p* < 0.01). The genetic correlation between SF and TS was also significantly less than unity (*Z* = 3.50, *p* < 0.01).

### 3.4. Genetic Gain

EBVs increased substantially in 2022 for all traits compared to those in 2021 (Table 5). Relative genetic gains in the 2022 generation were: TS (488%), MSI (366%), SF (348%), AS (284%), SS (265%), and NE1 (246%).

## 4. Discussion

### 4.1. Non-Spawning Rates Under SPF Conditions

Across two consecutive generations (2021–2022), 55.95–61.28% of females failed to spawn during the 48-day annual observation period. This proportion is higher than the 30–51% reported for live-feed systems [6,29,30] and the 36% observed in fast-growth strains reared under identical conditions [8]. Four interrelated factors may explain this disparity:

First, micronutrient limitation may play a role. Natural diets enhance gonadal development substantially [31], with *Nereididae* eliciting the strongest effects on ovarian maturation. In 30-day feeding trials where live feeds (polychaetes or squid) constituted the primary diet, 30–51% of females failed to spawn [6,30]. Although our formulated diet (56% crude protein, 11% crude lipid, supplemented with fresh-chilled squid) satisfies basic amino-acid and fatty-acid requirements, key micronutrients essential for ovarian development—namely docosahexaenoic acid (DHA) and phosphatidylcholine, both indispensable for vitellogenin synthesis and final oocyte maturation [8,32]—may be insufficient. Consequently, some females may remain arrested at the previtellogenic stage.

Second, tank versus pond environment affects spawning performance. Females reared in cement tanks with 75% daily water exchange exhibited a mean SF of only 0.78–0.80, compared with 1.44–1.70 in earthen ponds [14,33,34]. Pond systems provide natural benthic biofilms, diel fluctuations in micronutrients, and broader spectral light cues, all of which stimulate ovarian development [34,35]. While SPF conditions eliminate pathogens, they may inadvertently remove some maturation triggers.

Third, genotype-specific trade-offs likely contribute. The breeding nucleus simultaneously selects for WSSV resistance and rapid growth. Disease-resistant lines commonly reallocate resources from gametogenesis to immune function, a pattern supported by Ren [30], who reported a positive correlation between SF and body weight, and by the observation that fast-growing strains typically achieve larger body sizes than disease-resistant strains at the same age. Under identical maturation conditions, our disease-resistant population displayed a higher proportion of non-spawning females than fast-growing populations [7,8]. Although the present study did not quantify this relationship specifically, the combined effect of size differences and resource reallocation likely explains the elevated incidence of non-spawning females observed in the disease-resistant strain.

Fourth, nocturnal observation bias may affect our estimates. Because all reproductive metrics were derived from manual daytime checks, we cannot exclude the possibility that some females ovulated or spawned overnight. Consequently, the proportion of non-spawning females may be overestimated and the total spawn count underestimated. Such omissions likely occur randomly and involve both high-yielding and non-spawning females. Therefore, as in previous studies, we categorize this as a systematic error. Accurate reproductive phenotyping hinges on developing and deploying continuous, photoperiod-linked monitoring systems.

Collectively, the elevated non-spawning rate reflects a complex interplay among lipid micronutrients, environmental maturation cues, and genotype-specific resource allocation. Future work should (i) optimize feed formulation, (ii) evaluate temporary pond conditioning of SPF broodstock, and (iii) deploy continuous photoperiod-linked spawning monitors to refine phenotypic records.

### 4.2. Coefficient of Variation

In previous studies on shrimp spawning traits, coefficients of variation (CV) for SF and NE ranged from 35% to 93.4% [9,15,30,36]. These values are lower than those observed in the present study. A key reason for this discrepancy is that prior studies typically used live feeds (e.g., squid, polychaetes) for broodstock maturation. Under such conditions, broodstock gonads develop amply and replenish nutrients rapidly after spawning, facilitating quick entry into the next reproductive cycle. In contrast, the pellet feed and fresh-chilled squid used in this study were inferior to live feeds in promoting maturation. Only a portion of females achieved sufficient gonadal development. This outcome represents a compromise between achieving higher reproductive rates and controlling environmental pathogen risks. Consequently, substantial individual variation emerged in broodstock reproductive traits, reflected in the elevated CV values. Under these circumstances, the larger CV primarily reflects genuine biological heterogeneity rather than issues related to statistical models or experimental design.

Following one year of selective breeding, the 2022 broodstock exhibited significantly reduced CVs for SF, AS, TS, and SS compared to their parental generation. This suggests that, in addition to the targeted breeding traits, the SPF feed regimen also imposed selective pressure on reproductive performance. The selected broodstock demonstrated enhanced adaptability to this nutritional formulation.

### 4.3. Heritability

Heritability estimates for reproductive traits in the breeding nucleus were 0.19–0.36 for SF in 2021–2022. These moderate levels are higher than previously reported values (0.06–0.15) for *P. vannamei* [9,30,36]. This difference may be attributed to distinct experimental conditions. For instance, Ren [30] used a full live-feed regimen under normal seawater salinity, whereas Tan [9,36] applied a similar diet in brackish water. Under full live feeding, female shrimp generally exhibited more complete gonadal development, and genetic differences among families were likely smaller. In contrast, under our formulated-feed conditions, maturation-promoting effects varied more significantly among families. Our results therefore suggest that the capacity for multiple spawning under formulated feed conditions is heritable and amenable to genetic improvement. Comparative evidence from other species supports this interpretation. Yoshida [37] reported a heritability of 0.53 for SF in *O. niloticus*, which exceeds the values estimated for *P. vannamei* in the current study. Conversely, Gonçalves [16] reported heritability estimates ranging from 0.15 to 0.17, which are lower than our observations.

The TS heritability observed in 2021 (0.28) is comparable to estimates of 0.28–0.39 in rainbow trout [38,39,40,41]. In 2022, heritability dropped to 0.19, which falls within the range of 0.09–0.26 documented previously in *P. vannamei* [9,13,15,30,36]. These results indicate that egg-production traits can be effectively enhanced through selective breeding without detectable deterioration in female condition or offspring quality across consecutive spawns within a single generation [42].

Comparative analyses across species reveal consistently low heritability for MSI (0.06–0.22). In *O. niloticus*, MSI heritability ranges from 0.02 to 0.12 [16], whereas Tan [36] reported 0.10 ± 0.03 in brackish-water *P. vannamei.* Our two-generation dataset yielded a similar estimate of 0.10 ± 0.04, confirming that MSI is a reproductive-timing trait characterized by conserved low additive genetic variance ($h^{2}$ < 0.15) across aquatic taxa.

The heritability of SS in 2021 was higher than the 0.32 value reported by Tan [9]. This elevated value may be attributed to the inclusion of a portion of newly introduced population from 2019 as broodstock in 2020, thereby enhancing genetic diversity. The 2022 estimate was lower than that of the same study. In *O. niloticus*, SS heritability was 0.14 [37], whereas Trọng [43] reported a range of 0.17–0.22 over a 32-day period. The heritable nature of SS is supported by the following evidence: the mean spawning rate per family increased from 46.5% in 2021 to 60.46% in 2022, and all families successfully spawned in 2022 (Table 2). This variation among families suggests that SS can be improved through selective breeding.

This study provides the first quantitative-genetic characterization of two additional reproductive traits in *P. vannamei*: NE1 and AS. The heritability of NE1 was 0.33 ± 0.10 in 2021 but declined to 0.06 ± 0.05 in 2022. Similarly, AS exhibited moderate heritability in 2021 (0.40 ± 0.10) but decreased to a low level in 2022 (0.10 ± 0.06). In *O. niloticus*, Gonçalves [16] reported AS heritability ranging from 0.08 to 0.18. The 2021 estimate in this study is higher than that for *O. niloticus*, while the 2022 value is comparable. This pattern suggests that strong selection pressure had a more pronounced effect on these two traits.

To ensure consistency, we recorded the first spawning event from the start of the formal observation period, even though minimal spawning occurred in a small number of females beforehand. These early spawners constituted an insignificant fraction of the total sample.

The rapid reduction in heritability for all reproductive traits in the 2022 population was primarily driven by intense directional selection. A high proportion of non-spawning females failed to contribute offspring, thereby eroding additive genetic variance. During the 2021 mating process, 151 families provided 532 female broodstock (Table 1). Among these, 326 females were eliminated naturally due to failure of ovarian maturation (Figure 2). Even under controlled inbreeding, only 39 out of 206 females with mature ovaries successfully produced offspring. This resulted in a broodstock selection rate of merely 7.33%. The high selection intensity significantly reduced additive genetic variation for reproductive traits, leading to the substantial decline in heritability estimates in 2022.

Furthermore, convergence failure occurred when common environmental effects were included in the animal model. This was attributable to (i) limited numbers of phenotyped females per generation and (ii) the single-sire nested mating design, where paternal identities could only be assigned at the virtual-family level. Consequently, additive genetic effects were partially confounded with common environmental influences, potentially inflating the reported heritability estimates.

### 4.4. Genetic Correlations

Genetic correlations between SF and MSI, NE1, AS, and TS were strongly positive, ranging from 0.82 to 0.98 (*p* < 0.01). These results align with previous studies [30] and are biologically plausible, indicating that these reproductive traits are genetically linked through common regulatory mechanisms. MSI exhibited near-perfect positive correlations with NE1 (0.99 ± 0.18), AS (0.94 ± 0.15), and TS (0.99 ± 0.08).

These exceptionally strong correlations have key practical implications. Since these traits are genetically nearly synonymous, breeders can achieve comprehensive genetic improvement across multiple reproductive dimensions by focusing selection pressure on just one or two practically measurable traits, such as SF and TS. This streamlined approach reduces the complexity and cost of breeding programs while ensuring more stable production and enabling higher productivity with fewer broodstock females.

### 4.5. Genetic Gain

Our study demonstrates significant improvement in reproductive traits (246–488% one generation) in the breeding population, although these traits were not direct selection targets. To our knowledge, this represents the first report of genetic progress in reproductive traits in a shrimp breeding program not focused on reproduction.

Two key factors contributed to this unexpected progress. First, the high proportion of non-spawning females (>55%) created strong unintentional selection pressure, allowing only highly fecund individuals to reproduce. This effectively made the feeding regime an additional selection agent. Second, the natural mating system (as opposed to artificial insemination) enabled reproductively superior individuals to better express their potential, produce more viable offspring, and successfully pass these advantageous traits to the next generation.

These findings suggest that unconscious selection for reproductive traits within the breeding system can significantly influence phenotypic expression. This is particularly evident in SPF conditions, where diet-induced maturation can rapidly select for broodstock adapted to the breeding regime, even when reproductive traits are not explicitly targeted. Our results highlight the need to comprehensively consider the impact of breeding systems on broodstock performance.

## 5. Conclusions

In practice, a small number of rapidly developing females may mature and spawn before the formal establishment of families. Although these sporadic early events could not be accurately recorded, their impact is negligible due to the small number of individuals involved. The high heritability estimates for reproductive traits in 2021 indicated considerable potential for genetic gain. Although overall heritability declined in 2022, the results confirm these traits remain heritable and suitable as selection criteria. Notably, despite not explicitly targeting reproductive traits in our selection protocol, measurable genetic gain was observed for each trait. The mating design inadvertently prioritized highly fertile females as broodstock. These findings demonstrate that incorporating reproductive performance as a breeding objective could yield substantial improvements in hatchery efficiency without compromising other economic traits.

## Figures and Tables

**Table 1:** Numbers of females, sires, dams, full-sib and half-sib families by generation.

Generation	Females (n)	Sires (n)	Dams (n)	Full-Sib Families (n)	Half-Sib Families (n)
2021	532	150	130	151	50
2022	454	41	39	47	18
Total	986	191	169	198	68

**Table 2:** Descriptive statistics of reproductive traits for female *P. vannamei*.

Year	Traits (Unit)	Mean	Minimum	Maximum	SD	CV%
	SF (n)	0.80	0.00	5.00	1.24	156.4
	MSI (d)	2.30	0.00	35.00	5.31	230.53
2021	NE1 (10^4^)	4.83	0.00	31.00	7.12	147.46
	AS (10^4^)	5.04	0.00	31.00	7.17	142.33
	TS (10^4^)	10.30	0.00	115.00	17.36	168.52
	SS (%)	46.05	8.30	100.00	0.24	51.32
	SF (n)	0.78	0.00	6.00	1.14	145.73
	MSI (d)	2.37	0.00	47.00	6.03	254.7
2022	NE1 (10^4^)	6.94	0.00	36.00	8.59	123.75
	AS (10^4^)	6.89	0.00	41.00	8.76	127.12
	TS (10^4^)	11.26	0.00	82.50	15.57	138.32
	SS (%)	60.46	16.67	100.00	0.24	39.63

Note: SS values represent spawning success for each family. SF: spawning frequency, MSI: mean spawning interval, NE1: number of eggs laid for the first time, AS: average spawning, TS: total spawning, SS: spawning success.

**Table 3:** The variance components and heritability estimates for SF, MSI, NE1, AS, and TS within and across generation.

Year	Trait	$\sigma_{P}^{2}$	$\sigma_{a}^{2}$ **/** ${4\sigma }_{sd}^{2}$	$\sigma_{e}^{2}$	$h^{2}$
	SF	1.51	0.55	0.96	0.36 ± 0.08
	MSI	28.12	6.09	22.03	0.22 ± 0.08
2021	NE1	51.08	16.75	34.33	0.33 ± 0.10
	AS	52.23	20.94	31.29	0.40 ± 0.10
	TS	302.08	85.01	217.07	0.28 ± 0.10
	SS	1.06	0.26	0.80	0.79 ± 0.12
	SF	1.31	0.25	1.06	0.19 ± 0.08
	MSI	36.45	2.26	34.19	0.06 ± 0.05
2022	NE1	73.90	4.49	69.41	0.06 ± 0.05
	AS	76.83	7.32	69.51	0.10 ± 0.06
	TS	243.48	43.44	200.04	0.18 ± 0.07
	SS	1.00	0.03	0.97	0.12 ± 0.08
	SF	1.44	0.43	1.01	0.30 ± 0.06
	MSI	31.92	3.33	28.59	0.10 ± 0.04
ALL	NE1	61.18	7.56	53.62	0.12 ± 0.05
	AS	63.32	9.70	53.62	0.16 ± 0.06
	TS	277.54	66.93	210.61	0.28 ± 0.07
	SS	1.03	0.12	0.91	0.23 ± 0.06

SF: spawning frequency, MSI: mean spawning interval, NE1: number of eggs laid for the first time, AS: average spawning, TS: total spawning, SS: spawning success.

**Table 4:** Genetic correlations (top diagonal) and phenotypic correlations (bottom diagonal) of the traits studied over two successive generations.

Trait	SF	MSI	NE1	AS	TS
SF	—	0.98 ± 0.09	0.83 ± 0.11	0.82 ± 0.10	0.93 ± 0.02
MSI	0.60 ± 0.02	—	0.99 ± 0.18	0.94 ± 0.15	0.99 ± 0.08
NE1	0.64 ± 0.02	0.37 ± 0.03	—	0.99 ± 0.00	0.99 ± 0.05
AS	0.64 ± 0.02	0.39 ± 0.03	0.96 ± 0.00	—	0.98 ± 0.04
TS	0.91 ± 0.00	0.58 ± 0.02	0.77 ± 0.01	0.80 ± 0.01	—

Note: SF: spawning frequency, MSI: mean spawning interval, NE1: number of eggs at first spawning, AS: average spawning, TS: total spawning.

**Table 5:** Genetic progression estimated using mean EBV of two generations.

Trait	EBV		Genetic Progress	Percentage (%)
	2021	2022		
SF	0.03	0.12	0.09	348
MSI	0.08	0.35	0.28	366
NE1	0.07	0.24	0.17	246
AS	0.08	0.29	0.21	284
TS	0.16	0.94	0.78	488
SS	0.01	0.05	0.04	265

Note: SF: spawning frequency, MSI: mean spawning interval, NE1: number of eggs at first spawning, AS: average spawning, TS: total spawning, SS: spawning success.

## Data Availability

The original contributions presented in this study are included in the article; further inquiries can be directed to the corresponding author.
